# Squared nasal root, nasal voice -indicators of 22 q11.2 deletion in patients with psychiatric illness

**DOI:** 10.1186/1755-8166-7-S1-P76

**Published:** 2014-01-21

**Authors:** Jyothilakshmi Annavarapu, Prabhavathi Halappa, Niby J Elackatt, Mitesh Shetty, Sridevi Hegde

**Affiliations:** 1 Dept. of Medical Genetics, Manipal Hospital, Bangalore, India

**Keywords:** 22q11.2 deletion, DGS, Schizophrenia

## Background

22q11.2 Deletion Syndrome DGS (22q11) is a micro deletion syndrome caused by the deletion on chromosome 22. It is a multi system disorder which affects Cardiovascular system, immune system, facial features covered by acronym CATCH22 (Cardiac defects aortic arch anomalies, conotruncal anomalies, ventricular septal defect, patent ductus arteriosis and tetra logy of fallot; Abnormal facies ; Thymic hypoplasia; Hypocalcemia). Few children will not present with all of the above clinical features but only delayed motor mile stones, learning disability and mild behavioral issues which may progress onto psychiatric illness in adulthood. In this study, we aim to study the prevalence of DGS in patients with psychiatric illness.

## Materials and methods

To further explore physical, behavioral and psychiatric findings associated with 22q11 deletion in adults with psychiatric illness, we assessed 12 patients. All were confirmed psychiatric cases referred from a well-known Institute for mental health studies and also had mild facial dysmorphism. All patients were screened using the clinical checklist for DGS and Fluorescence In Situ Hybridization(FISH) studies was conducted using the probe specific for *TUPLE1* gene located on chromosome 22 q11.2 in cultured blood samples.

## Results

Out of 12 cases, six cases (50 %) tested positive for 22q11 deletion indicating a strong association between 22q11.2 deletion syndrome and psychiatric illness in adult population. All six patients presented with squared nasal root, and nasal voice in addition to psychosis and one also had cardiac abnormality (VSD). Recent studies report that Digeorge critical region (DGCR) spanning up to 2 Mb on chromosome 22q11 region contains several genes like *TBX1*, *GNB1L*, *PRODH* and *ZDHHC8* which are strong candidate genes for schizophrenia susceptibility. In conclusion, all patients with psychiatric illness, squared nasal root and nasal voice should be investigated for DGS.

